# Theranostic π Electron‐Stabilized Polymeric Micelles for PET Imaging‐Guided Drug Delivery to Tumors

**DOI:** 10.1002/smll.74543

**Published:** 2026-07-30

**Authors:** Alec Wang, Alexandru Florea, Jan‐Niklas May, Zaheer Ahmed, Karolin Roemhild, Thomas Hansen, Rahaf Mihyar, Armin Azadkhah Shalmani, Laura Schäfer, Karolin Becker, Masoud Sadeghzadeh, Eva M. Buhl, Sven Thoröe‐Boveleth, Susanne Lütje, Agnieszka Morgenroth, Fabian Kiessling, Jochen Maurer, Josbert M. Metselaar, Yang Shi, Felix M. Mottaghy, Quim Peña, Twan Lammers

**Affiliations:** ^1^ Institute For Experimental Molecular Imaging Uniklinik RWTH Aachen Aachen Germany; ^2^ Department of Nuclear Medicine Uniklinik RWTH Aachen Aachen Germany; ^3^ Department of Radiology and Nuclear Medicine Maastricht University Medical Center Maastricht The Netherlands; ^4^ Institute of Gynecology and Obstetrics Uniklinik RWTH Aachen Aachen Germany; ^5^ Electron Microscopy Facility Institute of Pathology Uniklinik RWTH Aachen Aachen Germany; ^6^ Institute for Occupational Social and Environmental Medicine Uniklinik RWTH Aachen Aachen Germany; ^7^ Center of Integrated Oncology Aachen Bonn Cologne Duesseldorf (CIO ABCD) Cologne Germany

**Keywords:** biodistribution, cancer research, chemistry, drug delivery, in vivo, micelle, nanomedicine, paclitaxel, positron emission tomography

## Abstract

Nanomedicines are increasingly employed in oncology. However, their efficacy is limited by heterogeneous nanoparticle accumulation in different tumors and patients. Nuclear imaging offers non‐invasive, patient‐ and lesion‐specific visualization and quantification of nanoparticle uptake, providing a biomarker to predict nanotherapy efficacy. We present a positron emission tomography (PET)‐imageable [mPEG‐*b*‐*p*(HPMAm‐Bz)]‐based polymeric micelle platform for image‐guided and tumor‐targeted drug delivery, with potential for patient stratification and theranostics. Polymers with 1 or 3 deferoxamine (DFO) chelators were synthesized, and the corresponding micelles showed stable ^89^Zr‐radiolabeling and efficient drug (paclitaxel) encapsulation. PET imaging revealed long in vivo circulation times and high tumor accumulation, with more DFO per polymer accelerating drug release and increasing off‐target accumulation. Hence, single‐DFO‐containing polymers were used to develop companion diagnostic and paclitaxel‐loaded theranostic micelles. Both formulations displayed comparable biodistribution profiles and high levels of tumor uptake (>17% ID/g) and were able to capture inter‐ and intra‐individual heterogeneity in tumor targeting. Importantly, cryo‐preservation enables long‐term storage while maintaining in vivo performance and tumor‐targeting capabilities, bolstering translational potential. Taken together, DFO‐functionalized π‐electron‐stabilized micelles allow direct quantification of nanoparticle accumulation in tumors while mediating effective drug delivery, showing promise for patient stratification and as a theranostic platform for image‐guided and personalized cancer therapy.

## Graphical abstract

Cancer nanomedicine efficacy is limited by heterogeneous nanoparticle accumulation in tumors. Nuclear imaging offers non‐invasive, patient‐ and lesion‐specific visualization and quantification of nanoparticle uptake. We present a positron emission tomography (PET)‐imageable micelle platform for direct quantification of nanoparticle accumulation and distribution in tumors, while mediating effective drug delivery. The theranostic platform opens opportunities for patient stratification and image‐guided cancer therapy.

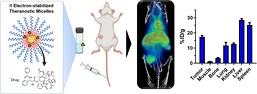

## 1 Introduction

Nanoparticles are extensively explored for drug delivery, particularly in cancer therapy. They improve drug administration and modulate biodistribution, reducing off‐target accumulation and promoting drug uptake in vascularized, inflamed, and pathological tissues such as tumors [1]. Together, these features enhance the efficacy and/or reduce the side effects of chemotherapy, as observed with the clinical nanoformulations Doxil [2] (liposomal doxorubicin) and Abraxane [3] (protein‐bound paclitaxel). However, while more than 15 nanomedicines have been approved for cancer treatment to date, the success rate for clinical translation of nanomedicines remains low relative to the large volume of publications on novel nanoformulations [4, 5].

The efficacy of cancer nanomedicines is often associated with their accumulation in tumors. This has been traditionally attributed to the enhanced permeability and retention (EPR) effect [6] and, more recently, also the active transport and retention (ATR) effect [7, 8]. In general, nanoparticles accumulate in tumors due to a combination of passive vascular leakiness and active transport, as well as abnormal lymphatic drainage and tumor‐associated macrophage uptake, promoting nanoparticle retention. However, the tumor uptake of nanomedicines has proven to be highly variable across different types of cancers and between patients [1]. Therefore, strategies to monitor and predict the tumor accumulation of nanomedicines over time are needed to promote nanomedicine clinical translation.

Biomarkers have emerged as a relevant tool for characterizing tumor physiology, stratifying patients and individualizing therapy regimens for improved treatment outcomes. Many anticancer drugs encompassing antibody therapeutics, kinase and checkpoint inhibitors already stratify patients based on biomarker expression, both during their clinical trials and as part of routine therapy management. This can improve therapeutic outcomes by selecting and treating the patients who are more likely to respond [9]. Yet, there are currently no biomarker protocols established for cancer nanomedicines. In particular, imaging biomarkers have attracted attention due to their ability to accurately and non‐invasively quantify (nano)drug accumulation, providing high specificity while imposing relatively low burden on the patient [10]. This has also allowed for theranostic approaches, where a single system enables both diagnostic and therapeutic opportunities [11]. Nuclear imaging techniques such as positron emission tomography (PET) and single photon emission computed tomography (SPECT) are highly valuable for in vivo theranostic applications, due to their high sensitivity and specificity [12].

Nanoparticle platforms are particularly suited to theranostics, as they can be readily functionalized for non‐invasive imaging at the same time as mediating drug delivery [13, 14, 15]. This makes them attractive as imaging biomarkers for improving cancer nanomedicine performance. They can provide individualized information on the uptake of nanoformulations into tumors, thereby allowing for the identification and/or pre‐stratification of the patients that are most likely to benefit from a specific nanotherapy [9]. This has already been explored and demonstrated in clinical trials, as a retrospective study that correlated nanotherapy response to pre‐therapeutic nuclear imaging [16]. However, such nanotheranostic constructs can bring about translational challenges, often due to their complex formulation design and low manufacturing feasibility, which can limit eventual pharmaceutical development [17].

Taking all of the above into account, we set out to develop an imageable polymeric micelle platform for image‐guided drug delivery and cancer nanotheranostics. The polymeric micelles are constituted by biocompatible PEGylated HPMA‐based block co‐polymers, self‐assembled and stabilized through hydrophobic and π‐π interactions [18]. These micelles have shown high capacity for hydrophobic drug delivery, with promising preclinical performance when loaded with anticancer drugs such as paclitaxel (PTX) and docetaxel, while being compatible with scale‐up and continuous‐flow manufacturing protocols [19, 20, 21]. To enable their use as an imaging biomarker, we functionalized the platform with a metal chelator (deferoxamine, DFO) to allow for radionuclide‐based PET imaging and quantitative assessment of tumor targeting, in addition to the delivery of hydrophobic drugs. In this study, we first optimized the radiometal‐chelating micelle platform by systematically testing different degrees of DFO‐functionalization per polymer for particle stability, and we assessed the labeling efficiency with ^89^Zr and the in vivo PET imaging performance. Then, we explored its feasibility as a nanotheranostic platform by integrating ^89^Zr as a diagnostic marker and PTX as a chemotherapeutic drug, and we analyzed their impact on the pharmaceutical properties of the formulations. Finally, we tailored the micelles for image‐guided drug delivery, comparing the tumor accumulation and in vivo imaging performance of different nanoformulation designs to evaluate their potential use as an imaging biomarker as well as for theranostic applications.

## 2 Materials and Methods

### 2.1 Materials and Reagents

[^89^Zr]Zr(IV)‐oxalate was purchased from Perkin‐Elmer LAS (Germany) and/or Monrol (Turkey). [^18^F]FDG was ordered from Advanced Accelerator Applications Germany GmbH (Germany). Deferoxamine (DFO) mesylate was purchased from Sigma‐Aldrich (USA) and paclitaxel (PTX) from MP Biomedicals (Germany). Rhodamine–conjugated ricinus communis agglutinin I RL‐1082‐1 (Rhodamine‐Lectin) was sourced from Vector Laboratories (USA). All other reagents, including solvents, were acquired from standard commercial suppliers and used as received. The starting materials for the polymer synthesis; *N*‐(2‐benzoyloxypropyl) methacrylamide (HPMAm‐Bz monomer) and (mPEG_5k_)_2_‐4,4′‐azobis(4‐cyanopentanoic acid) (macroinitator) were synthesized based on reported procedures [21].

### 2.2 DFO‐Functionalized Polymer Synthesis

#### 2.2.1 Polymer Precursor Synthesis

**FIGURE 1:**
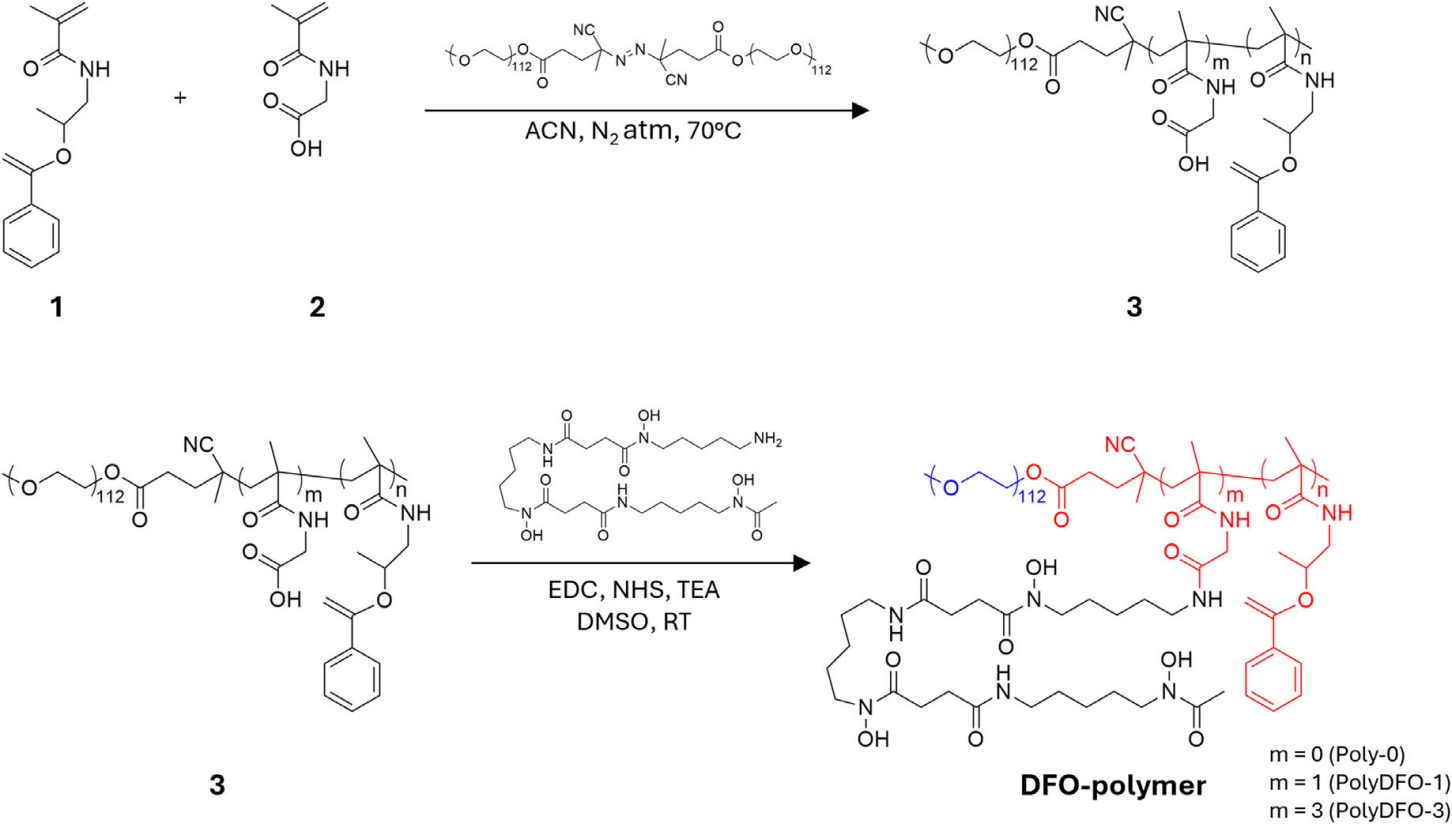
Synthesis of deferoxamine (DFO) chelator‐functionalized polymers. Synthesis of [PEG‐*b*‐p(HPMAm‐Bz)]‐based block co‐polymers by free radical polymerization including post‐functionalization with DFO chelators. For the final polymer (DFO‐polymer): in blue, the hydrophilic block (PEG), in red the hydrophobic block (HPMAm‐Bz and MAG monomeric units), and in black, the DFO chelator conjugated to the hydrophobic backbone of the block co‐polymer via an amide bond. The ratio of DFO per polymer (m) varies between 0 (Poly‐0), 1 (PolyDFO‐1), or 3 (PolyDFO‐3), while the degree of polymerization of HPMAm‐Bz monomers (n) is preserved around 70 (*n* = 70).

Precursor block co‐polymers PEG‐*b*‐p[(MAG)_m_‐*co*‐(HPMAm‐Bz)_n_] (with n ca. 70, and m = 1 or 3) (Figure 1, Compound 3) were synthesized by free radical polymerization. HPMAm‐Bz (Figure 1, Compound 1), *N*‐methacryloylglycine (Figure 1, Compound 2, MAG) and the macroinitiator [(mPEG_5k_)_2_‐4,4’‐azobis(4‐cyanopentanoic acid)] were dissolved at a 200:2X:1 molar ratio in acetonitrile (ACN) at a HPMAm‐Bz concentration of 300 mg/mL (NB: X corresponds to the target number of DFO chelator units per polymer chain, with 0 for Poly‐0, 1 for PolyDFO‐1 and 3 for PolyDFO‐3). The reaction vessel was degassed by nitrogen purge for at least 15 min, and the polymerization reaction was carried out at 70°C for 20 h under a nitrogen atmosphere. The resulting product was precipitated in diethyl ether (50 mL), washed with additional diethyl ether (x2, 50 mL), and dried in vacuo to yield an off‐white powder. The product was characterized via proton nuclear magnetic resonance (^1^H NMR; Varian AV400 and AV600, Bruker Corporation, USA) and gel permeation chromatography (GPC; 1260 II Infinity LC system, Agilent Technologies, USA), following similar protocols as previously reported [22].

PolyDFO‐1 precursor PEG‐*b*‐p[(MAG)_1_‐*co*‐(HPMAm‐Bz)_70_] – Precipitation yield: 73%. Mn (GPC): 16 kDa, Đ 1.5.

PolyDFO‐3 precursor PEG‐*b*‐p[(MAG)_3_‐*co*‐(HPMAm‐Bz)_70_] – Precipitation yield: 75%. Mn (GPC): 16 kDa, Đ 1.9.

#### 2.2.2 Chelator Functionalization

DFO functionalization of the precursor polymers PEG‐*b*‐p[(MAG)_m_‐*co*‐(HPMAm‐Bz)_70_] (n = 1 or 3) was performed via EDC/NHS coupling between DFO and the precursor, forming an amide bond between the MAG co‐monomer unit and the DFO (Figure 1, Compound 4). The precursor polymer was dissolved in dimethyl sulfoxide (DMSO) at a concentration of 150 mg/mL. The precursor polymer was activated by the addition of a 20% excess of *N*‐hydroxysuccinimide (NHS) and 1‐ethyl‐3‐(3‐dimethylaminopropyl)carbodiimide (EDC), and the pH was adjusted to 6 by addition of triethylamine (TEA). After 1 h under stirring, a 20% excess of DFO was added along with additional TEA to raise the pH to 8, and the reaction was allowed to proceed overnight at room temperature. The reaction mixture was purified by dialysis (7 kDa MWCO SpectraPor, Repligen, USA) against DMSO for three cycles and finally dried in vacuo overnight to yield an off‐white powder. The polymers were dissolved in DMSO‐*d_6_* and characterized by 400 MHz ^1^H NMR (Varian AV400 and AV600, Bruker Corporation, USA). The NMR spectra were processed and analyzed in MestReNova 6.0 (Mestrelab Research S.L., Spain). The degree of DFO functionalization was measured by integration of the peak at 7.75 ppm corresponding to the hydroxamic acid groups of DFO. The sample was also characterized by GPC (1260 II Infinity LC system, Agilent Technologies, USA) under the same conditions as Section 2.2.1.

PolyDFO‐1 Polymer PEG‐*b*‐p[(MAG‐DFO)_1_‐*co*‐(HPMAm‐Bz)_70_] – Precipitation yield: 74%. Mn (GPC): 16 kDa, Đ 1.5.

PolyDFO‐3 Polymer PEG‐*b*‐p[(MAG‐DFO)_3_‐*co*‐(HPMAm‐Bz)_70_] – Precipitation yield: 72%. Mn (GPC): 17 kDa, Đ 1.9.

*Summary of polymer terminology and nomenclature*. Three different polymers were used in this study to develop micelle formulations. These were differentiated by the average number of DFO chelator units per polymer chain. **PolyDFO‐1** and **PolyDFO‐3** are [PEG‐*b*‐p(HPMAm‐Bz)]‐based polymers containing 1 and 3 conjugated DFO units on average per chain, respectively, while **Poly‐0** has no DFO units.

### 2.3 Preparation of Polymeric Micelles

Polymeric micelles were produced following a similar nanoprecipitation protocol to previous work [18]. For fully DFO‐functionalized micelles (PM1 and PM3) and non‐functionalized micelles (PM0), 30 mg of the corresponding polymer (Poly‐0 for PM0, PolyDFO‐1 for PM1 or PolyDFO‐3 for PM3) were dissolved in 1 mL tetrahydrofuran (THF). For blended micelles (Blend‐PM), Poly‐0 and PolyDFO‐1 polymers were mixed in a 9:1 ratio (w/w), and 30 mg of total polymer were dissolved in 1 mL of THF. For drug‐loaded formulations, 7.5 mg of PTX were added to the polymer‐THF solution. The THF solution was then added dropwise to 1 mL of Milli‐Q water under stirring at 1000 rpm, and stirring was maintained for 2 min. The solutions were then left at room temperature for at least 20 h to evaporate THF, leaving an opalescent dispersion of polymeric micelles. For cryo‐preservation of micelles (Cryo‐PTX‐Blend‐PM), a similar protocol was followed as previously reported [19]. In short; during micelle preparation, the Milli‐Q water was replaced by a 8% (w/w) sucrose solution. The sucrose‐containing micelle dispersion was then snap‐frozen in liquid nitrogen after THF evaporation, followed by storage at ‐20°C until use. The cryo‐preserved micelles (Cryo‐PTX‐Blend‐PM) were reconstituted as needed by thawing at room temperature and filtration through a 0.2 µm nylon syringe filter (Whatman, Sigma Aldrich, Germany) before use.

#### 2.3.1 Summary of Polymeric Micelle Terminology and Nomenclature

**PM0** micelles are made from Poly‐0, therefore containing no DFO chelator units. **PM1** and **PM3** are purely made from PolyDFO‐1 and PolyDFO‐3 polymers, respectively, with 1 and 3 DFO chelators on average per polymer in the micelle. **Blend‐PM** is a micelle made of a 9:1 mix of Poly‐0 and PolyDFO‐1 polymers. When loaded with paclitaxel, “PTX‐” is added as a prefix. Additionally, micelles that were cryo‐preserved are marked with the “Cryo‐” prefix. Therefore, Cryo‐PTX‐Blend‐PM is a cryo‐preserved, paclitaxel‐loaded polymeric micelle made of a 9:1 blend of Poly‐0 and PolyDFO‐1 polymers.

### 2.4 Physicochemical Characterization

The size and size distribution of the micelles were characterized by dynamic light scattering (DLS; Zetasizer Nano‐ZS, Malvern Panalytical Ltd., UK) and transmission electron microscopy (TEM; Hitachi HT7800, Hitachi, Japan). For DLS, 20 µL of the micelle dispersion (30 mg/mL) was diluted 50x in Milli‐Q water, and 1 mL was transferred into a disposable polystyrene cuvette. Samples were measured with a fixed 173° backscatter angle at 25°C using automatic software control over attenuation. For TEM, micelle dispersions (30 mg/mL) were diluted 200x in Milli‐Q water, adsorbed onto formvar‐carbon‐coated nickel grids (Maxtaform 200 mesh, Electron Microscopy Sciences, USA) and washed 3x with Milli‐Q water. The samples were negatively stained with 0.5% uranyl acetate (Science Services GmbH, Germany) and imaged. Critical micelle concentration (CMC) was measured by the standard coumarin fluorescence method, following reported procedures [23]. The stability of the particles was measured by diluting the micelles 1:3 in phosphate‐buffered saline (PBS) and storing them in the fridge at 4°C for 1 month. The size and PDI of the particles were then measured by DLS to assess any changes during the storage period. Likewise, the stability in physiological conditions was measured by diluting the micelles 1:3 in fetal bovine serum (FBS) at pH 7.4 and then incubating the mixture at 37°C with gentle agitation for 96 h. The size and PDI were then measured by DLS to determine stability.

### 2.5 Drug Loading and Retention

Paclitaxel (PTX)‐drug encapsulation was measured by HPLC (1260 II Infinity LC system, Agilent Technologies, USA) after nanoparticle disruption in acetonitrile, using a previously reported method [22]. PTX drug retention in the micelles was measured in sink conditions, also following a previously reported protocol [22]. In brief, the micelle formulations were incubated in a 45 mg/mL bovine serum albumin (BSA)‐containing PBS solution at pH 7.4 in a dialysis device (Float‐a‐Lyzer, Spectrum Labs, USA) under agitation for 72 h at 37°C. Samples (50 µL) were taken at different time points (24, 48 and 72 h) from inside the dialysis device and 450 µL of ACN was added to disrupt the micelles and precipitate the BSA. The sample was then centrifuged, and the supernatant containing the encapsulated PTX was measured by HPLC using a similar protocol as used for PTX encapsulation.

### 2.6 Radiolabeling and Characterization

Radiolabeling of all polymeric micelles was conducted with the same protocol. Polymeric micelles were prepared as described in Section 2.3. Approximately 100 MBq of [^89^Zr]Zr‐oxalate in 1 M oxalic acid was neutralized with 2 M Na_2_CO_3_ for 3 min before 1.8 mL of 0.05 M HEPES buffer and 0.2 mL of micelle dispersion (30 mg/mL of polymer) were added. The reaction was allowed to proceed for 90 min at room temperature. The mixture was then transferred to a centrifugal filter (Amicon Ultra‐4, 10 kDa preconditioned by 100 µL PBS; Merck Millipore Ltd, Ireland) and purified by centrifugation at 4000 rpm for 25 min to reach a volume of ca. 100 µL. Then, 900 µL PBS (pH 7.4) was added to the filter and centrifuged again at 4000 rpm for 25 min to afford the formulated ^89^Zr‐labeled micelles in PBS. Micelle stability under this protocol was assessed using cold Zr(IV) oxalate in place of [^89^Zr]Zr‐oxalate, with samples taken every 15 min and the size and size distribution measured by DLS. The product was then assessed for radiochemical purity by RTLC (Mini Gita‐Beta Detector GMC; Raytest GmbH, Germany) using iTLC‐SG strips and Citrate buffer eluent as well as drug‐loading by HPLC. Radiolabel stability was measured by incubating the labeled micelle formulations in PBS or fetal calf serum at 37°C, pH 7.4. Aliquots (50 µL) were taken at different time points (24, 48 and 96 h), and radiochemical purity was measured by RTLC following the method previously established. Drug release assessment was conducted as specified above.

### 2.7 Animal Model Care and Handling

The animal experiments were performed in accordance with the Guide for the Care and Use of Laboratory Animals and were approved by the governmental animal welfare authorities (LANUV, AZ 84‐02.04.2017.A045, AZ 81‐02.04.2023.A089). Four‐ to eight‐week‐old female BALB/c mice (Charles River, Germany; used in Biodistribution study 1, see Section 2.8), 4–6 weeks old female NOD/SCID mice (Janvier, France; used in Biodistribution study 2, see Section 2.8) and 6–8 weeks old female BALB/c mice (Janvier, Germany; used in Biodistribution study 3, see Section 2.8) were housed in groups of 5 under specific pathogen‐free, temperature and humidity‐controlled conditions. Water and standard food pellets (Sniff GmbH, Germany) were available at libitum.

4T1 triple‐negative breast cancer (TNBC) was chosen as a prototypic mouse tumor model, since it is syngeneic, well‐established, and the most widely used preclinical tumor model in nanomedicine research [24]. 4T1 cells (American Type Culture Collection, Manassas, VA, USA) were cultured in Roswell Park Memorial Institute (RPMI) medium (RPMI 1640; Gibco, Life Technologies GmbH, Germany), supplemented with 10% fetal bovine serum (Life Technologies GmbH, Germany) and 1% penicillin/streptomycin (10000 U/mL penicillin; 10 mg/mL streptomycin) (Life Technologies GmbH, Germany). The mice were inoculated orthotopically with 1 × 10^5^ 4T1 cells in the left mammary fat pad of the female BALB/c mice to establish a TNBC model.

BCSC‐1 was chosen as a patient‐derived TNBC model that could reproduce a more complex tumor microenvironment to assess the performance of the formulations [25]. To establish the model, a small sagittal incision (approximately 1 cm) was made on the shaved and sterilized abdomen that permits access to the #4 mammary gland on both sides. 1 × 10^5^ BCSC‐1 cells were mixed with 1 × 10^6^ irradiated human fibroblasts (NuFF, MTI‐GlobalStem, USA) in 40 µL per gland of MSC medium and Matrigel (Corning, USA) at a 1:1 ratio. The seeding mixture was injected into the mammary fat pad on both sides of the NOD/SCID female mice. Each transplant was localized distal to the lymph node in the gland. Surgical incisions were sealed by suturing with a 5/0 thread.

For both models, tumors were allowed to grow until they reached 7–11 mm (based on caliper measurements) in one of the dimensions before being randomly allocated to a treatment group. Animals were assessed for possible metastasis during the course of the experiment by inspection of PET/CT imaging. No indications of metastatic spread to the common sites of 4T1 metastasis, including the lungs, bone, or lymph nodes, were observed. This is consistent with previous experience with this model, where metastasis does not develop until 5 weeks after tumor induction [26].

### 2.8 In Vivo Biodistribution

#### 2.8.1 Biodistribution Study 1

*In vivo imaging of radiolabeled micelles*. 4T1 tumor‐bearing mice (Charles River) were randomized into the following groups (N = 4 per group): i) [^89^Zr]Zr‐DFO, ii) [^89^Zr]Zr‐PM1, or iii) [^89^Zr]Zr‐PM3. Anesthesia was induced by inhalation of 5% isoflurane under oxygen and maintained by adjusting isoflurane content to maintain a respiratory rate of 60 breaths per minute. The mice in the [^89^Zr]Zr‐PM1 and [^89^Zr]Zr‐PM3 groups were injected with 5 MBq of [^18^F]FDG intravenously via the tail vein under anesthesia and imaged by dynamic PET/CT from 0 to 45 mins post‐injection at 5 min per frame (Triumph II, Northridge Tri‐Modality Imaging, Inc., USA). 24 h later, all animals were injected with 5 MBq of the assigned agent; [^89^Zr]Zr‐DFO, [^89^Zr]Zr‐PM1 or [^89^Zr]Zr‐PM3, intravenously via the tail vein. Both micelle groups were co‐administered with PM0 micelles up to a total polymer mass injected of 100 mg/kg in 100 µL. PET/CT images were acquired at 4, 24, 48 and 72 h postinjection (Triumph II). The mice were sacrificed after the 72‐h scan. Excised organs were cut into 8 µm thick sections, and the radioactivity in the sections was imaged by autoradiography (Typhoon FLA 7000, GE Healthcare, USA).

CT acquisition settings: magnification 1.3, 130 µA, 75 kVp, 230 ms exposure time, and 360° rotation with 720 views with an average of 2 frames for each view; the duration of the CT scans was approx. 15 min. A dynamic 45 min PET scan was initiated at the end of the CT scan. The CT had an axial field of view of 91.1 mm, while the PET had one of 112 mm. The CT images were reconstructed using a Feldkamp filtered back projection reconstruction process to a voxel size of 0.154 × 0.154 × 0.154 mm^3^ in a 592 × 592 × 560 matrix. Using vendor software, the CT values were converted into Hounsfield units (HU) using the following formula:

$$HU=1000\times (\left(\mu_{t}-\mu_{w}\right)\;/\;\left(\mu_{w}-\mu_{a}\right)$$

where µ_w_ is the linear attenuation coefficient of water, µ_a_ is the linear attenuation coefficient of air, and µ_t_ is the linear attenuation coefficient of the tissue. The PET data were reconstructed using a 3D ordered‐subset expectation maximization (i.e., OSEM‐3D with 3 iterations and 8 subsets) with a maximum *a posteriori* probability algorithm (30 iterations) into a 240 × 240 × 192 image matrix (resulting in final voxel dimensions of 0.25 × 0.25 × 0.597 mm^3^). PET normalization, CT‐based attenuation correction, and CT‐based scatter correction were applied during all PET reconstructions. PET and CT images were automatically aligned after reconstruction using the matrix generated from a capillary phantom scan. Organ segmentation was performed manually and quantified as mean target‐to‐background (tibialis anterior muscle) ratios (TBR_mean_) for comparison between groups using PMOD Version 4.4 (PMOD Technologies LLC, Switzerland). Blood half‐life was estimated using PET quantification of the blood volume of the left ventricle of the heart as a surrogate measure for the blood pool. In short, a spherical volume of interest of 1 mm radius was placed in the center of the ventricle, and the PET signal was quantified in that volume. GraphPad Prism software version 10 (GraphPad Software, USA) was used to estimate blood half‐life (t_1/2_) by fitting a one‐phase exponential decay curve by the least‐squares method.

#### 2.8.2 Biodistribution Study 2

*Comparison of biodistribution in a patient‐derived xenograft model*. BCSC‐1 patient‐derived triple negative tumor‐bearing mice were administered 5 MBq of [^89^Zr]Zr‐PM1 intravenously via the tail vein under anesthesia. The mice were co‐administered with PM0 micelles intravenously via the tail vein up to a total polymer mass injected of 100 mg/kg in 100 µL. After 48 h, the mice were sacrificed, and total uptake of [^89^Zr] in the individual organs was measured ex vivo by a gamma counter (Wizard2, PerkinElmer, USA). Excised organs were cut into 8 µm thick sections, and the radioactivity in the sections was imaged by autoradiography. To maintain comparability to the 4T1 model, the tumor in the left flank was used for analysis.

#### 2.8.3 Biodistribution Study 3

*In vivo imaging of drug‐loaded radiolabeled micelles (theranostic formulations)*. 4T1 tumor‐bearing mice were randomized into three groups (*n* = 6 per group) and administered 5 MBq and 15 mg/kg PTX of one of three micelle formulations intravenously via the tail vein under anesthesia. These formulations were: i) *PTX‐Mono*, comprised of ^89^Zr‐radiolabeled PM1 micelles co‐administered with PTX‐loaded PM0 micelles, ii) *PTX‐Blend*, comprised of ^89^Zr‐radiolabeled PTX‐Blend‐PM micelles or iii) *PTX‐Cryo*, comprised of ^89^Zr‐radiolabeled Cryo‐PTX‐Blend‐PM micelles (Figure 5A). Anesthesia was induced by inhalation of 5% isoflurane under oxygen and maintained by adjusting isoflurane content to maintain a respiratory rate of 60 breaths per minute. All micelle groups were co‐administered with additional nondrug‐loaded PM0 micelles up to a total polymer mass injected of 100 mg/kg in 100 µL to ensure comparable polymer concentration in all cases. PET/CT imaging was acquired 15 min and 24 h postinjection (β‐CUBE, and X‐CUBE from Molecubes NV, Belgium). 15 min before sacrifice, the animals were injected (intravenously via tail vein) with 60 µL of 5 mg/mL rhodamine–conjugated ricinus communis agglutinin I (Rhodamine‐lectin) to label perfused blood vessels for ex vivo fluorescence imaging.

CT acquisition settings: Standard high‐resolution protocol, 440 µA, 50 kVp, 32 ms exposure time, and 1080° rotation in a spiral mode with 960 exposures/360°; the duration of each CT scan was approx. 5 min. The CT axial field of view is 37 mm. A dynamic 45 min‐PET scan was initiated at the end of the CT scan. The PET axial field of view is 160 mm. CT images were reconstructed using an iterative reconstruction algorithm (i.e., ISRA) reconstruction process to an isometric voxel size of 0,2 mm in a 200 × 200 × 400 matrix. Using vendor‐provided software, CT values were converted into Hounsfield units (HU). PET data were reconstructed using a 3‐dimensional ordered‐subset expectation maximization (i.e., OSEM‐3D with 30 iterations) with an energy window of ±15% from a peak of 511 keV to an isometric voxel size of 0,4 mm in a 192 × 192 × 384 matrix. CT‐based corrections (i.e., attenuation correction, scatter correction, dead time, and random events) were applied during the PET reconstruction. PET and CT images were automatically aligned after reconstruction using the matrix generated from a capillary phantom scan. Organ segmentation was performed manually and quantified as %ID/g (normalized to individual organ weight) to compare nanocarrier accumulation and drug delivery between formulations using PMOD Version 4.4 (PMOD Technologies LLC, Switzerland).

*Summary of theranostic micelle formulation terminology*. **PTX‐Mono** is a formulation containing ^89^Zr‐radiolabeled PM1 micelles mixed with PTX‐PM0 micelles. **PTX‐Blend** is a formulation comprised of only ^89^Zr‐radiolabeled PTX‐Blend‐PM micelles; and **PTX‐Cryo** is made up of ^89^Zr‐radiolabeled PTX‐Blend‐PM micelles with added sucrose to ensure cryo‐protection of the formulation at −20°C.

### 2.9 Ex Vivo Tumor Analysis

After sacrifice, tissues were excised and embedded in Tissue‐Tek OCT (Sakura, USA) at ‐80°C before being cut into 10 µm thick cryosections. The sections were fixed on glass slides using 80% (v/v) methanol in water at room temperature, followed by acetone at −20°C. Immunofluorescence staining was carried out in a humidified chamber in the dark. Rat anti‐F4/80 (Abcam, UK) primary antibodies were diluted (1:50) in 12% (w/v) BSA and applied for 1 h at room temperature, followed by washing with phosphate‐buffered saline (PBS) to remove excess antibodies. Alexa Fluor 488 anti‐rabbit secondary antibody (1:350; Dianova, Germany) together with 4′,6‐diamidino‐2‐phenylindole (DAPI) nuclei staining (Merck, Germany) diluted 1:500 in 12% BSA were applied for 45 min at room temperature, followed by another washing step. The stained sections were then mounted with Mowiol 4–88 (Carl‐Roth, Germany) and covered with a glass coverslip. Fluorescence microscopy images were acquired using an AxioImager M2 microscope with an AxioCam MRm Rev.3 camera (Carl Zeiss AG, Germany). Representative images with a were acquired from the core (*n* = 5) and periphery (*n* = 5) per tumor slice at a magnification of 100 using the following exposure times; 9 µs for DAPI and 700 µs for both Rhodamine‐Lectin and F4/80. The area fraction (%) of the respective signals was quantified with CellProfiler version 4.2.8 (Broad Institute, USA).

### 2.10 Statistical Analysis

Data are presented as value ± standard deviation unless otherwise specified. Data and statistical analysis were performed using GraphPad Prism version 10. One‐way ANOVA or two‐way ANOVA with multiple comparisons with Bonferroni corrections were applied as appropriate for the dataset in each case. *p*‐value thresholds for significance: *p* > 0.05 (ns), *p* < 0.05 (*), *p* < 0.01 (^**^), *p* < 0.001(^***^).

## 3 Results

### 3.1 Deferoxamine‐Functionalized Polymer and Micelle Preparation

First, we set out to determine the optimal number of DFO units per polymer for theranostic applications, maintaining maximum particle, drug, and radiolabel stability while also providing high labeling yield and adequate imaging performance. For that purpose, we synthesized [mPEG‐*b*‐p(HPMAm‐Bz)]‐based block co‐polymers with 0, 1, or 3 DFO units per polymer chain and systematically evaluated the impact of the DFO density on the properties of the corresponding micelle formulations. To minimize the effect of the DFO functionalization on the nano‐biological interactions, the chelator was incorporated in the core of the micelles (by derivatizing the hydrophobic block of the polymer), and not on the PEG‐shell. The synthetic protocol to obtain the DFO‐conjugated polymers was based on post‐polymerization functionalization of the polymer scaffold precursor (compound 3) with 1 (PolyDFO‐1) or 3 (PolyDFO‐3) DFO units, through an amide bond formation (Figure 1). The reaction produced an off‐white powder for both PolyDFO‐1 and PolyDFO‐3 (precipitation yield of ca. 70%). Based on ^1^H NMR and GPC characterization, both PolyDFO‐1 and PolyDFO‐3 had comparable polymer features (Mn of 16–17 kDa and Đ of 1.5‐1.9), and the target number of DFO units on average per polymer chain (Figure S1).

**FIGURE 2:**
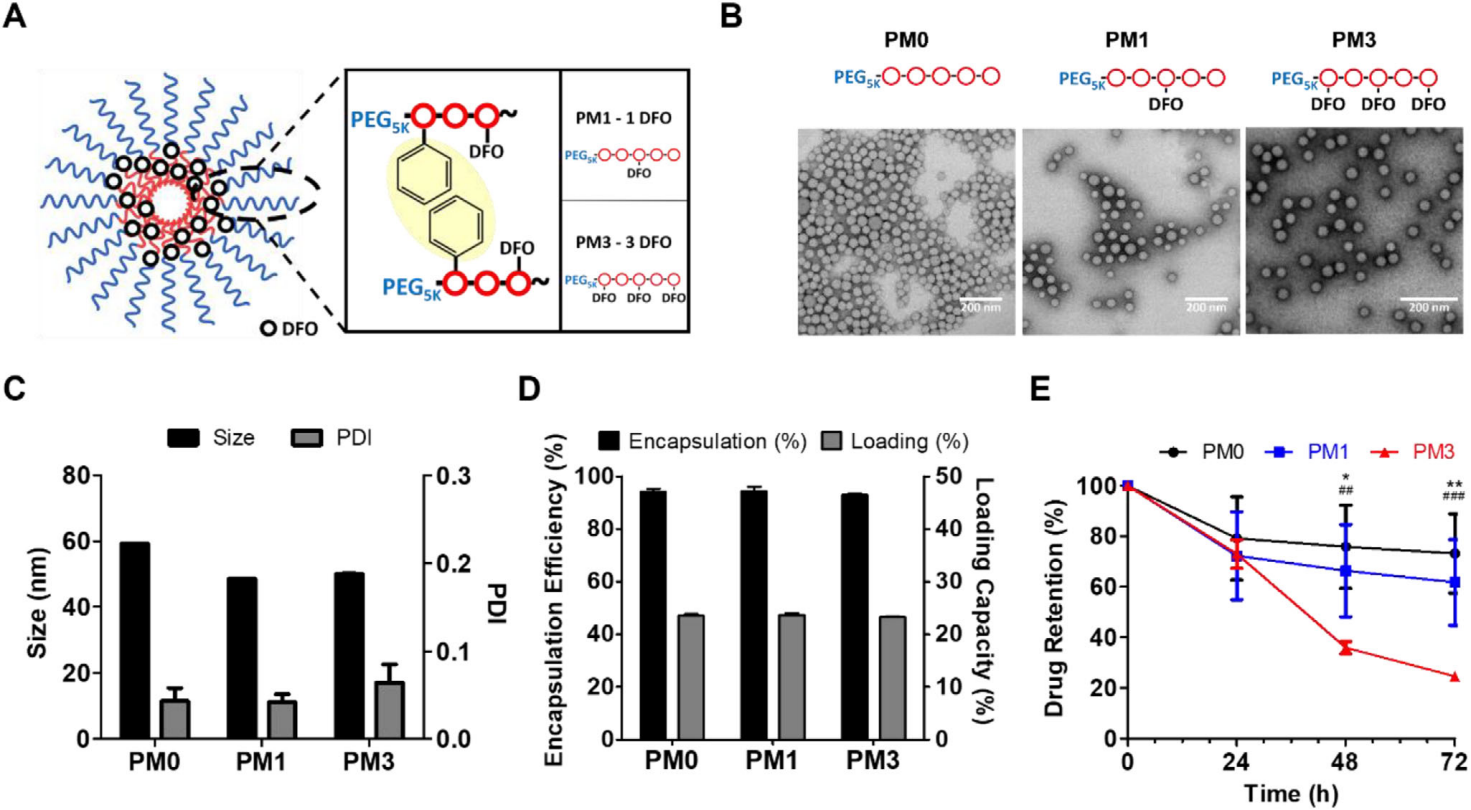
Formulation and characterization of DFO‐functionalized polymeric micelles. (A) Polymeric micelle structure showing π–π stabilization of the aromatic rings of the HPMAm‐Bz units in the hydrophobic core. The ratio of the average DFO units per polymer chain varies between 0 (PM0 micelles), 1 (PM1 micelles) or 3 (PM3 micelles). (B) Nondrug‐loaded micelle size and morphology by transmission electron microscopy (NB: the scheme of the constituting polymer for each micelle type is shown for clarity: i.e., Poly‐0 for PM0, PolyDFO‐1 for PM1 and PolyDFO‐3 for PM3). (C) Nondrug‐loaded micelle size (hydrodynamic diameter, Z_average_) and polydispersity (PDI) by dynamic light scattering (DLS), *N* = 3. (D) Paclitaxel drug‐encapsulation efficiency (EE) and loading capacity (LC) of the micelle formulations, *N* = 3. (E) Paclitaxel retention in the micelle formulations in physiological sink conditions (at 37°C in 45 g/L albumin‐containing PBS media at pH 7.4), * = statistical significance PM1 vs PM3, ^#^ = statistical significance PM0 vs PM3; *N* = 3 micelle formulations. Statistical analysis by two‐way ANOVA with Bonferroni correction: *p* < 0.05 (^*^), *p* < 0.01 (^**^), *p* < 0.001 (^***^).

The DFO‐functionalized PolyDFO‐1 and PolyDFO‐3 polymers were then used to form π electron‐stabilized polymeric micelles (Figure 2A). Polymeric micelles (PM1 and PM3) produced from both polymers were spherical in shape, with 50 nm particle diameter and a narrow size distribution (PDI < 0.1), in line with the non‐functionalized micelles (PM0) (Figure 2B,C). Additionally, micelles produced from both PolyDFO‐1 and PolyDFO‐3 polymers had comparable critical micelle concentrations (CMC) between them and with the non‐functionalized micelles PM0 (3.5–6 µg/mL), indicating similar stability upon dilution (Figure S2). The micelles were stable for at least one month when stored in pH 7.4 PBS at 4°C and for 96 h when incubated in pH 7.4 FBS at 37°C (Figure S3). The impact of DFO density on the pharmaceutical properties of the corresponding formulations was evaluated by loading the clinical chemotherapeutic paclitaxel (PTX), as a model hydrophobic drug, into the nonfunctionalized (PM0) and DFO‐functionalized micelles (PM1 and PM3). The drug encapsulation efficiency (EE) was very similar for all three polymeric micelles (EE values > 90%), indicating that the degree of DFO‐functionalization did not affect the high loading capacity of the system (Figure 2D). However, when drug release was evaluated in sink conditions and biologically relevant media (in albumin‐containing PBS at pH 7.4 and 37°C), the results showed that a higher degree of DFO functionalization resulted in a significantly lower drug retention capability of the micelle platform (Figure 2E).

### 3.2 Radiolabeling of the Micelle Formulations

**FIGURE 3:**
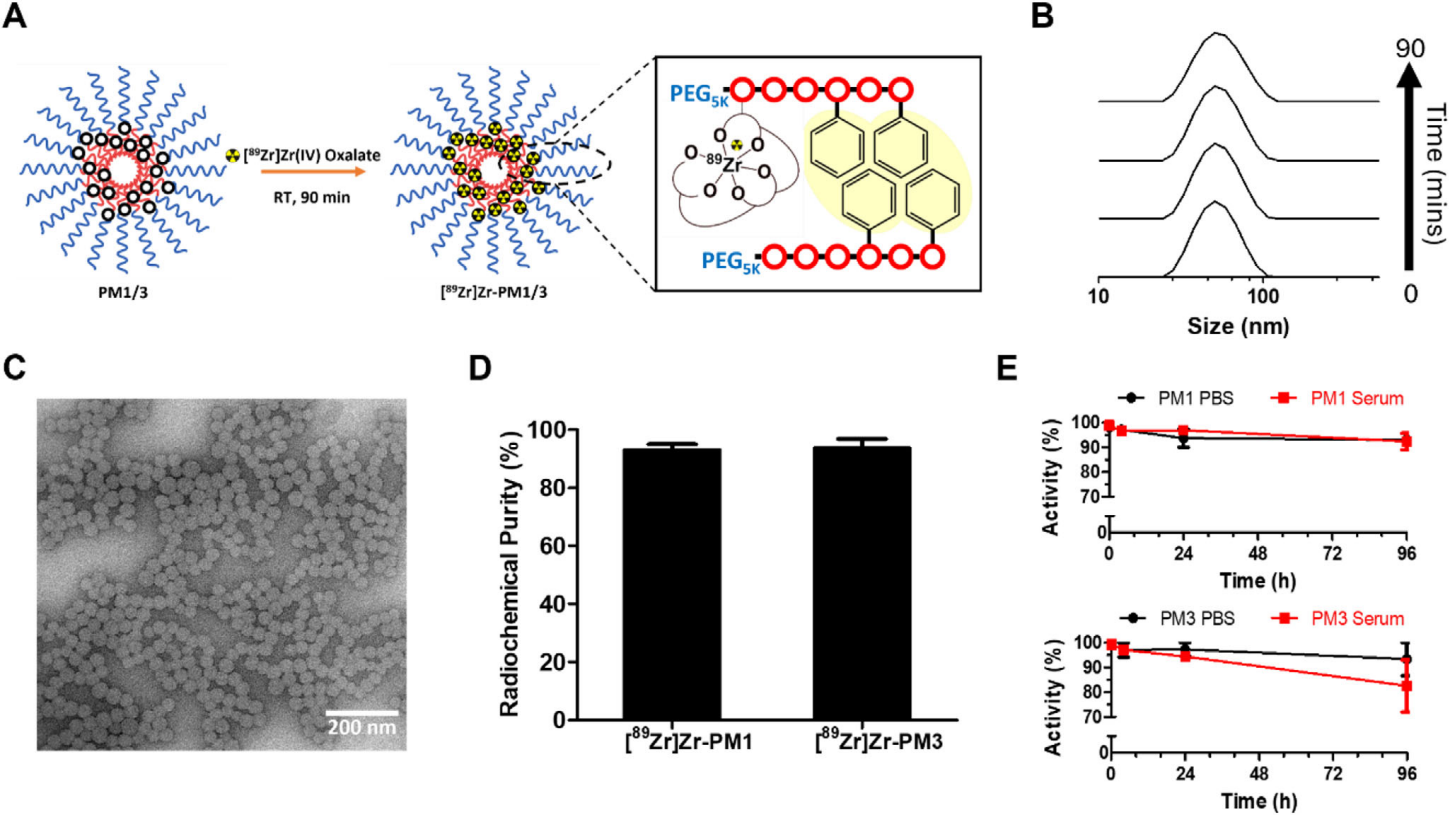
Radiosynthesis of ^89^Zr‐labeled polymeric micelles for PET imaging. (A) Chelation of ^89^Zr by DFO‐functionalized polymeric micelles, stabilized by π–π and hydrophobic interactions in the micelle core. (B) Polymeric micelle size distribution during the radiolabeling procedure using a nonradioactive Zr(IV) salt by DLS. Each band represents the size distribution at 0, 30, 60, and 90 min after addition of the Zr(IV) salt. (C) Size and morphology of radiolabeled polymeric micelles by TEM. (D) ^89^Zr‐labeling radio‐conversion and recovery by radio‐iTLC, *N* = 3. (E) Stability of ^89^Zr‐radiolabeling of PM1 (top) and PM3 (bottom) over time, incubated in PBS (pH 7.4) and serum at 37°C, *N* = 3 micelle formulations. Statistical analysis by two‐way ANOVA with Bonferroni correction: *p* < 0.05 (^*^), *p* < 0.01 (^**^), *p* < 0.001 (^***^).

Then, we labeled the DFO chelator‐functionalized micelles with a radionuclide for PET imaging (Figure 3A). Radiolabeling of nondrug‐loaded PM1 and PM3 micelles with ^89^Zr was carried out at room temperature using the corresponding oxalate salt, resulting in high radiochemical yields (70%) and purity (93%) after 90 min of reaction and after centrifugal purification (Figure 3D). Micelle properties remained stable during labeling, with no measurable change in size distribution or spherical shape, as confirmed by DLS and TEM (Figure 3B,C). Radiolabeled micelles ([^89^Zr]Zr‐PM1 and [^89^Zr]Zr‐PM3) retained over 90% and 82% of the ^89^Zr radionuclide, respectively, after 96 h in serum media at 37°C, indicating very high metal chelation stability, which is crucial for eventual in vivo imaging and theranostic applications (Figure 3E).

### 3.3 Impact of Polymer Functionalization Density on the In Vivo Imaging Performance

To study the effects of the polymer composition and degree of DFO chelator‐functionalization on the in vivo imaging performance of the micelle platform, we used a TNBC 4T1‐based mouse model. Orthotopic 4T1 TNBC tumor‐bearing mice were intravenously injected with either [^89^Zr]Zr‐PM1 or [^89^Zr]Zr‐PM3 micelles, as compared to free [^89^Zr]Zr‐DFO and [^18^F]FDG (as standard clinical PET probe for glucose metabolism in tumors) with a total activity of 5 MBq each. Since the total amount of nanoparticles has been reported to alter the biodistribution of nanomedicines [27], we homogenized the amount of particles across the groups by co‐administering them with additional non‐radiolabeled PM0 micelles, in order to reach a comparable total polymer dose of 100 mg/kg in each group.

**FIGURE 4:**
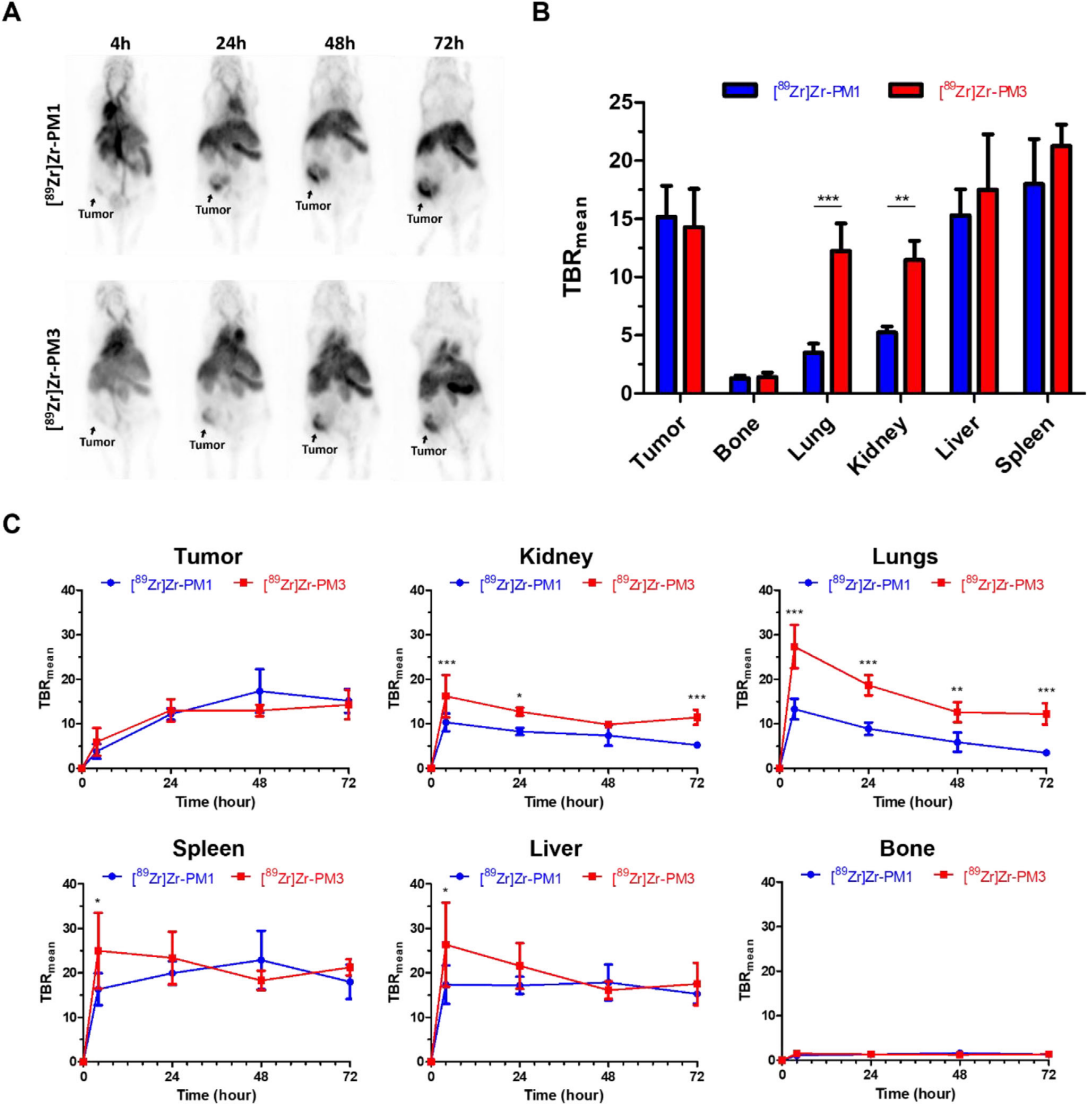
In vivo biodistribution and tumor accumulation of ^89^Zr‐labeled polymeric micelles. (A) Representative PET maximum intensity projections (MIP) for injected ^89^Zr‐labeled polymeric micelles PM1 and PM3 at 4, 24, 48, and 72 h post‐injection into orthotopic 4T1 tumor‐bearing mice. (B) Biodistribution and tumor accumulation of the polymeric micelles at 72 h post‐injection, comparing mean target‐to‐background (TBR_mean_, with muscle as the background). (C) [^89^Zr]Zr‐PM1 and [^89^Zr]Zr‐PM3 PET image contrast over time compared to background (muscle) for on‐target (tumor) and off‐target organs. *N* = 4 for each group. Statistical analysis by one‐way ANOVA (B) and two‐way ANOVA (C) with Bonferroni correction: *p* < 0.05 (^*^), *p* < 0.01 (^**^), *p* < 0.001 (^***^).

PET/CT imaging showed that the micelles had prolonged blood circulation half‐life and prominently accumulated in the tumor over at least 72 h, producing strong imaging contrast 24 h postinjection compared to the free [^89^Zr]Zr‐DFO chelator (Figure 4A; Figure S4). In contrast, [^18^F]FDG showed comparatively poor image contrast and tumor localization (Figure S6). [^89^Zr]Zr‐PM1 formulation produced the highest tumor contrast (TBR_mean_ of 17.4 ± 4.9 at 48 h at its peak) compared to [^89^Zr]Zr‐PM3 (TBR_mean_ 14.3 ± 3.3 at 72 h at peak), although without statistically significant differences between them (*p* > 0.05) (Figure 4B,C). Off‐target accumulation was similar between the two micelle formulations 72 h postinjection (Figure 4B), mainly in the liver and spleen, common off‐target organs for nanoparticles [28]. Importantly, the accumulation in the bone was very low for both micelle formulations as compared to the free [^89^Zr]Zr‐DFO (Figure 4B,C and Figure S5), confirming the high radiolabeling stability of the micelles in vivo.

[^89^Zr]Zr‐PM3 had a shorter blood half‐life compared to [^89^Zr]Zr‐PM1 (t_1/2_ of 24.5 vs. 28.5 h), indicating somewhat lower systemic stability and more rapid clearance. However, [^89^Zr]Zr‐PM3 accumulated significantly more than [^89^Zr]Zr‐PM1 in the kidneys (TBR_mean_ 11.5 vs. 5.2, *p* < 0.01) and lung (TBR_mean_ 12.2 vs. 3.5, *p* < 0.001) (Figure 4B,C), indicating that the DFO‐density in the constituting polymers (i.e., 1 vs. 3 DFO units per polymer) impacts the in vivo performance of the corresponding micelles (PM1 vs. PM3). From this, we concluded that 1 DFO per polymer chain (PolyDFO‐1 polymers) resulted in PM1 micelles more suited for imaging and theranostic applications, with strong imaging contrast available at 24 h postinjection (Figure S7), efficient tumor accumulation and the lowest off‐target accumulation, while at the same time ensuring adequate physicochemical and colloidal stability as well as drug retention capabilities (Figure 2). We then compared the performance of [^89^Zr]Zr‐PM1 micelles in a patient‐derived xenograft model of TNBC (BCSC‐1; Figure S8), showing an analogous biodistribution profile and high uptake in the tumors as observed for the 4T1 model (Figure 4). Ex vivo autoradiography confirmed the in vivo PET imaging data and demonstrated that the micelle platform can also capture the variability in the intratumoral distribution between and within individual lesions (Figure S9). Based on the good results in the above studies, we decided to proceed with the PolyDFO‐1 polymer configuration for further in vivo experiments and theranostic micelle formulation evaluation.

### 3.4 Preparation of Theranostic Micelle Formulations

**FIGURE 5:**
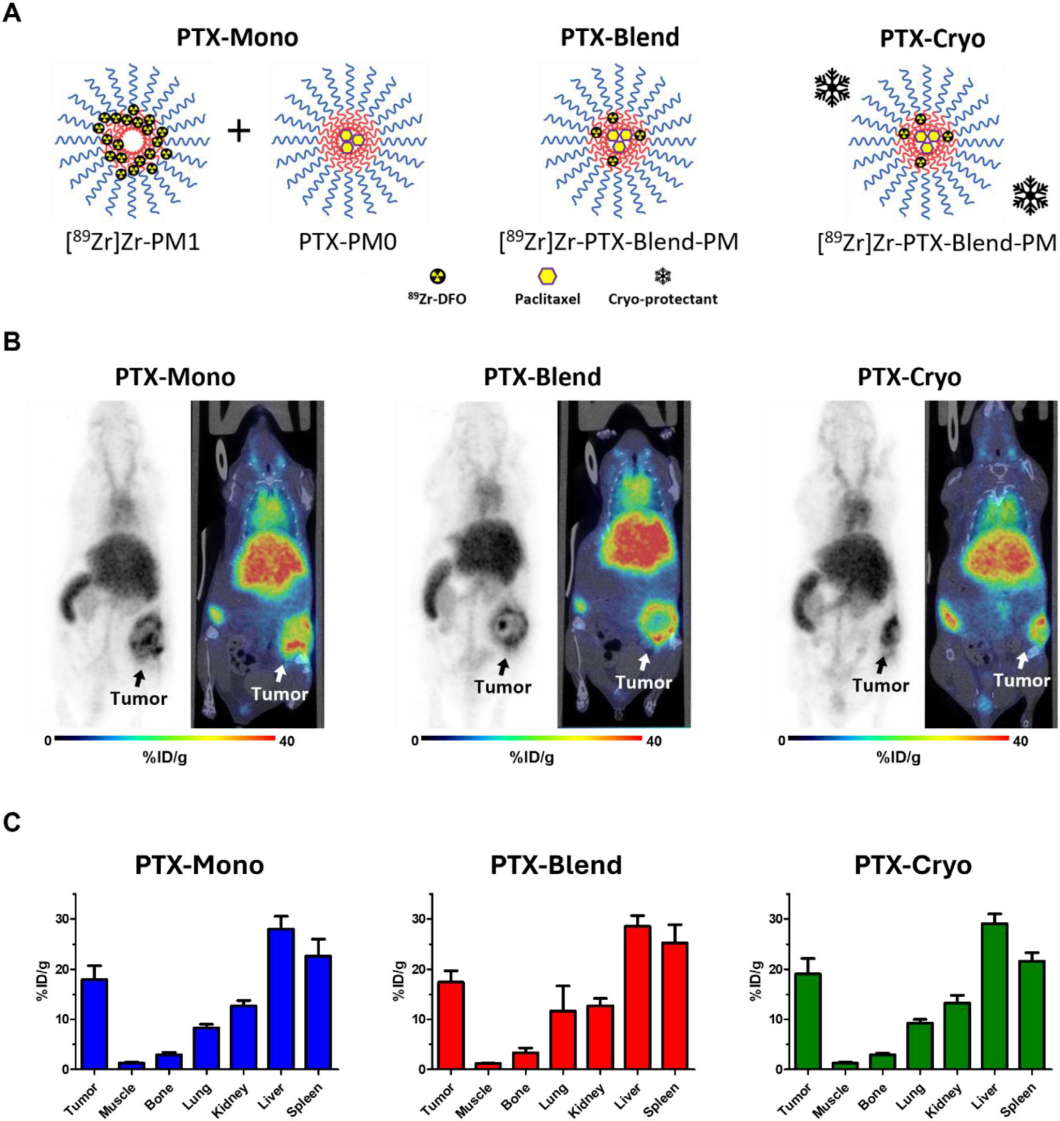
Biodistribution and tumor accumulation of theranostic micelle formulations. (A) Paclitaxel (PTX) drug‐loaded radiolabeled polymeric micelle formulation designs (PTX‐Mono, PTX‐Blend and PTX‐Cryo). (B) PET MIP (left) and PET/CT (right) of 4T1 tumor‐bearing mice at 24 h post‐intravenous injection of the theranostic formulations. (C) Tumor and organ accumulation normalized to organ weight (%ID/g) of the theranostic formulations at 24 h post‐injection by PET‐imaging. *N* = 6 for each group.

After selecting 1 DFO per polymer chain (PolyDFO‐1) for in vivo imaging applications of the micelle platform, we then set out to identify the most suitable formulation design for effective theranostic and image‐guided drug delivery applications. For that purpose, we developed two theranostic, PTX drug‐loaded micelle formulations: (a) *PTX‐Mono* (co‐formulating radiolabeled‐PM1 micelles, as a companion diagnostic, with unlabeled, PTX‐loaded PM0 micelles); and (b) *PTX‐Blend*, a formulation made of a single radiolabeled micelle type (PTX‐Blend‐PM) as a theranostic particle (Figure 5A). The PTX‐Blend‐PM micelles were also cryo‐preserved using sucrose as a cryo‐protectant and radiolabeled after thawing to yield the *PTX‐Cryo* formulation (Figure 5A). Effective cryo‐protection will enable long‐term storage and shipment of the theranostic formulation in frozen conditions (−20°C) [19], thus increasing their translational potential and eventual clinical applicability.

Results confirmed that the PTX drug‐loaded blended micelles (PTX‐Blend‐PM) were successfully produced by mixing Poly‐0 and PolyDFO‐1 polymers in a 9:1 (w/w) ratio with the drug PTX during the formulation, yielding nanoparticles with similar size distribution (about 55 nm and PDI < 0.1) and drug encapsulation efficiency (EE of 95% and LC of 23%) to the pure PM1 and to standard PM0 micelle formulation. PTX‐Blend‐PM micelles containing sucrose as cryo‐protectant were snap‐frozen and then thawed at room temperature before use, with minimal impact on size and preserving the homogeneity of the formulation (size around 70 nm and PDI < 0.1; Figure S10), as well as no drug loss (Figure S11).

Radiolabeling of the PTX‐loaded micelle formulations produced equivalent radiochemical yield and purity compared to the pure PM1 formulation (Figure S12). Drug release kinetics from the blended ^89^Zr‐labeled micelles ([^89^Zr]Zr‐PTX‐Blend‐PM) were similar to the PTX‐loaded ^89^Zr‐labeled pure PM1 micelles ([^89^Zr]Zr‐PTX‐PM1) and to unlabeled pure PM1 (between 50% and 60% drug retained after 72 h in all cases) (Figure S13 and 2E), indicating that blending and radiolabeling do not significantly accelerate drug release from the nanoparticles.

### 3.5 Comparative in Vivo Biodistribution Evaluation of Theranostic Micelles

Finally, we evaluated the in vivo performance of the three different theranostic formulation setups (PTX‐Mono, PTX‐Blend and PTX‐Cryo), to assess the impact on the biodistribution and tumor accumulation for eventual use in image‐guided cancer nanotherapy. Orthotopic 4T1 TNBC tumor‐bearing mice were divided into three groups and were intravenously injected with either the PTX‐Mono, PTX‐Blend, or PTX‐Cryo formulations at an equivalent PTX dose of 15 mg/kg and a total ^89^Zr activity of 5 MBq for each (Figure 5A). PET/CT imaging at 24 h postinjection showed similarly strong contrast, with a clear delineation of the tumor margin and necrotic core (Figure 5B). The three formulations achieved comparably efficient tumor accumulation, as confirmed by PET quantification, with all three resulting in high nanomedicine uptake at the tumor site (17.9 ± 2.8, 17.4 ± 2.3 and 19.1 ± 3.1%ID/g for PTX‐Mono, PTX‐Blend and PTX‐Cryo, respectively, *p* > 0.05) (Figure 5C). Off‐target accumulation was similar between all the groups (Figure 5C), with liver and spleen as the major off‐target organs. Very low signal was detected in the bone (< 5%ID/g with all three formulations), thereby confirming that the radiolabeling is also highly stable in vivo when the formulations are drug‐loaded. Nanomedicine tumor accumulation has been shown to be influenced by tumor microenvironment features such as tumor‐associated macrophage and perfused blood vessel density [29]. Ex vivo analysis of both macrophages and blood vessels in tumors showed that the three treatment groups had similar densities of both features, in line with the comparable tumor uptake observed (Figures S14 and S15 and Figure 5C).

**FIGURE 6:**
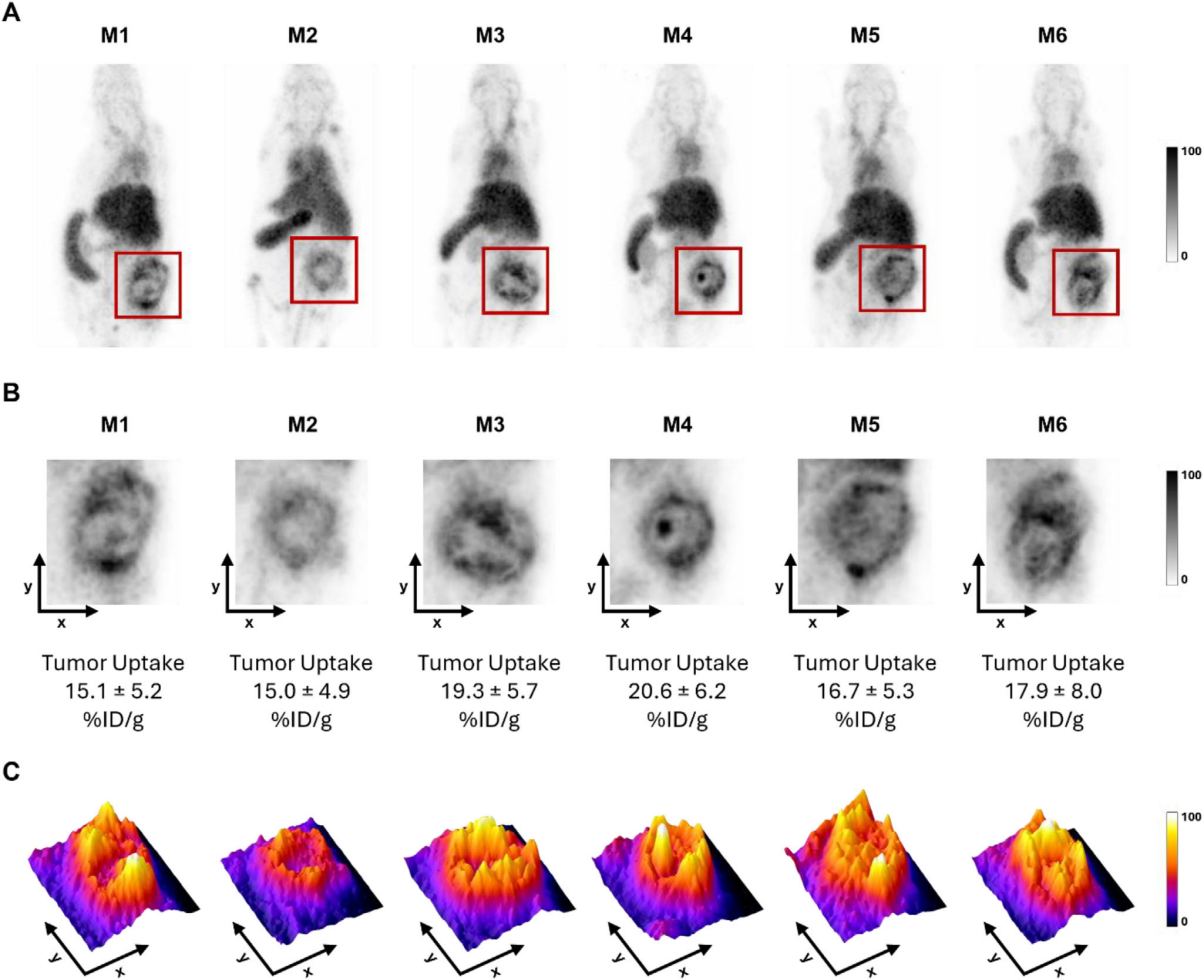
Imaging the heterogeneity of micelle uptake in tumors. (A) Individual PET MIP of 4T1 tumor‐bearing mice at 24 h post‐intravenous injection of PTX‐Blend micelles. (B) Zoom in MIP of the individual tumors, showing quantification of normalized micelle uptake in tumors (%ID/g ± standard deviation) and the variability between individuals. (C) The corresponding surface plots of normalized pixel intensity show the spatial intratumoral variation in micelle uptake within the individuals Scale bar:% of Max Intensity.

Finally, we analyzed the uptake and spatial distribution within the tumors using the theranostic nanoprobe (Figure 6). The micelles were able to quantify individualized tumor uptake and capture the heterogeneity between individual animals (Figure 6A,B). They additionally showed promise for mapping the variability of intratumoral nanoparticle uptake, revealing a heterogeneous spatial distribution, with the highest accumulation in the tumor margins and lower uptake in the core (Figure 6B,C). These data show that the imageable micelle platform is an effective vehicle for drug delivery to tumors while retaining their performance as an imaging agent, capturing the inter‐ and intra‐individual heterogeneity in tumor uptake and intratumoral distribution.

Altogether, we show that chelator density in the micelle core (below the pure PM1 threshold of one DFO unit per polymer in the micelle) does not influence the biodistribution and tumor accumulation of the nanoparticles, and that both PTX‐Mono and PTX‐Blend micelle formulations hold potential for tumor‐targeted theranostics. Meanwhile, cryo‐preservation can enable a practical means of storing and distributing the nanotheranostic platform for use in the clinic, without degrading its performance both as an imaging and as a drug delivery agent [19].

## 4 Discussion

We engineered an imageable polymeric micelle platform that can exploit quantitative nuclear imaging for eventual use as a companion diagnostic in cancer nanomedicine as well as for theranostic applications. This may address the challenges in the bench‐to‐bedside translation of nanomedicines, most prominently, the high variability in therapeutic responses due to cancer heterogeneity [30]. Imaging biomarkers provide high specificity for monitoring nanoparticle tumor accumulation and predict therapeutic efficacy, thereby enabling patient stratification to bolster the success rate in nanomedicine clinical translation and paving the way for improved treatment outcomes [9]. Optimization of the micelle platform was directed towards achieving high tumor accumulation (with minimal off‐target accumulation) while retaining drug payloads effectively, and with a view towards eventual nano‐pharmaceutical development and manufacturing.

Our findings demonstrate that increasing the density of the metal‐chelating agent DFO in the core of the micelles destabilizes the micelle formulation, lowering relative tumor uptake compared to other organs and resulting in a higher off‐target accumulation (particularly in the lungs) without improving imaging contrast or sensitivity (Figure 4). This could be related to in vivo aggregation of the micelles, since larger aggregates have been reported to be more readily entrapped in the fine capillary network of the lungs [31]. This instability may be due to disruptions of the hydrophobic and π‐π interactions in the micelle core in biological media, induced when more than one unit of the flexible and linear DFO chelator is introduced in the constituting polymers.

After identifying that one DFO unit per polymer chain was optimal for PET imaging contrast while preserving tumor targeting and drug delivery properties (Figures 3 and 4), two different formulation designs were conceived for imaging and theranostic applications (Figure 5). The first of these (PTX‐Mono) is envisaged as a companion diagnostic, in which the PET‐radiolabeled micelles (diagnostics) are co‐administered with unlabeled, PTX‐loaded micelles (therapy). The accumulation of nanomedicines can be evaluated on an individual level, potentially enabling patient stratification to promote clinical translation and improve nanotherapy outcomes [32]. The second formulation was focused on a theranostic approach, using the micelle platform for combined therapy (via drug delivery) and diagnostics (via PET/CT imaging) in a single particle (and, eventually, in a single formulation vial). Monitoring of disease progression and nanoparticle uptake during therapy using such theranostic or companion diagnostic formulations can enable faster and more accurate decision‐making compared to surrogate measures such as CT alone, while being less invasive and more direct than tissue biopsy [33, 34, 35]. Particularly in a neoadjuvant setting, the ability to identify the dynamics of nanoparticle accumulation over the course of therapy, or the optimal time for surgical resection, could improve treatment outcomes [36, 37].

Both PTX‐Mono and PTX‐Blend theranostic formulations showed comparably high tumor accumulation (>17% ID/g) and imaging contrast, with minimal off‐target uptake in the lungs, kidneys, or bone (Figure 5), thus highlighting the effectiveness of the developed polymeric micelle platform as a tumor‐targeted companion nano‐diagnostic and/or for nano‐theranostic applications. The micelle platform could capture the inter‐ and intra‐individual variation in tumor uptake of nanoparticles, as well as map the spatial heterogeneity of nanoparticle uptake within the tumor at high resolution (Figure 6). This allows for the identification of likely responders by their overall uptake as well as potentially imaging the development of the tumor. Compared to other nanomedicines reported in the literature (with an average of 0.7% ID/g of tumor uptake; reported in a meta‐analysis [38]), our polymeric micelles exhibit very high tumor accumulation, demonstrating the promise of this platform as a drug delivery system for cancer therapy [19, 39]. This performance likely stems from the high colloidal stability in vivo (due to the low CMC values) and minimal protein corona formation on the fully PEGylated nanoparticles [40], enhancing circulation time and reducing clearance by the reticular‐endothelial system [41]. Future studies will further explore the heterogeneity of nanoparticle uptake by expanding into other cancer models that may be more variable than 4T1, providing the opportunity to correlate uptake with tumor microenvironment factors. As a first step towards this, we showed that the micelles also performed well in a patient‐derived xenograft model, demonstrating similar biodistribution profiles, with the increased uptake heterogeneity expected of a more variable model (Figure S8 and S9). This will be further extended to therapeutic studies, where we aim to correlate individualized tumor nanoparticle accumulation with therapeutic efficacy.

From a translational perspective, the PTX‐Mono formulation may impose greater challenges while crossing the bench‐to‐bedside gap. It will require scale‐up production of two unique nanoparticle designs, a companion diagnostic micelle and a therapeutic micelle. This can result in a more complex pharmaceutical development; including considerations like long‐term stability and storage optimization, as well as quality control and regulatory evaluation for each particle design. On the other side, developing a single micelle platform that enables drug delivery as well as optional PET‐imaging when needed requires only a single particle type to be produced for both therapy and biomarker diagnostic applications. The successful cryopreservation of this formulation is a step forward towards demonstrating the feasibility of manufacturing for this micelle platform, enabling long‐term storage and distribution while preserving both imaging and drug delivery performance [19]. Likewise, imaging‐based patient stratification has the potential to increase the likelihood of achieving therapeutic responses efficacy, and it facilitates clinical trials, by optimizing therapy regimes and trial population sizes, together enhancing the translational potential of tumor‐targeted nanomedicine therapies [9]. Together, these features can streamline pharmaceutical development and regulatory pathways while also opening opportunities for in‐situ cancer nanotherapy monitoring and improved efficacy.

## 5 Conclusions

We developed a π‐electron‐stabilized PEGylated HPMA‐based polymeric micelle platform for image‐guided drug delivery and theranostic applications by functionalizing the constituting polymer scaffolds with different degrees of DFO chelator units per polymer chain. We showed that the resulting micelles were effectively radiolabeled with the PET radionuclide ^89^Zr with high radiolabeling stability, while also preserving drug encapsulation efficiency and retention capabilities. The micelle formulations showed high imaging contrast and tumor accumulation, enabling the localization of tumors and direct quantification of nanomedicine uptake. The optimal micelle platform for imaging was obtained with lower degrees of DFO functionalization in the core, which resulted in enhanced stability and significantly decreased off‐target accumulation. Finally, two different theranostic formulation designs were explored, in which the imageable micelle platform was either co‐administered with drug‐loaded micelles (i.e., as a companion diagnostic) or loaded with the drug in a single particle. Both formulations efficiently targeted tumors, including after cryopreservation for nanomedicine storage and distribution. These formulations retained the strong imaging performance of the nondrug‐loaded formulations, enabling the mapping of the spatial heterogeneity of nanoparticle uptake within the tumor as well as between individuals. These findings highlight the potential of the π‐electron‐stabilized polymeric micelle platform for tumor‐targeted and image‐guided drug delivery, with future application as an imaging biomarker for nano‐therapy monitoring as well as for cancer theranostics; together promoting nanomedicine clinical translation.

## Conflicts of Interest

The authors declare no conflicts of interest.

## Supporting information




**Supporting File**: smll74543‐sup‐0001‐SuppMat.docx.

## Data Availability

The data that support the findings of this study are available from the corresponding author upon reasonable request.
