# Supplementary material for: Theranostic π Electron‐Stabilized Polymeric Micelles for PET Imaging‐Guided Drug Delivery to Tumors
Source: Small. 2026 Jul 30;22(49):e74543. doi: 10.1002/smll.74543 (PMC13533258; doi:10.1002/smll.74543)
Supplement: Supplementary file 1 [file SMLL-22-e74543-s001.docx]

**- SUPPORTING INFORMATION -**

**Theranostic π electron-stabilized polymeric micelles for PET imaging-guided drug delivery to tumors**

Alec Wang^1^, Alexandru Florea^2,6^, Jan-Niklas May^1^, Zaheer Ahmed^1^, Karolin Roemhild^1^, Thomas Hansen^3^, Rahaf Mihyar^1^, Armin Azadkhah Shalmani^1^, Laura Schäfer^2^, Karolin Becker^2^, Masoud Sadeghzadeh^2^, Eva M. Buhl^4^, Sven Thoröe-Boveleth^5^, Susanne Lütje^2,7^, Agnieszka Morgenroth^2^, Fabian Kiessling^1^, Jochen Maurer^3^, Josbert M. Metselaar^1^, Yang Shi^1^, Felix M. Mottaghy^2,6,7^,

Quim Peña^1,*^, Twan Lammers^1,7,*^

^1^Uniklinik RWTH Aachen, Institute for Experimental Molecular Imaging, Forckenbeckstraße 55, 52074 Aachen, Germany

^2^Uniklinik RWTH Aachen, Department of Nuclear Medicine, Pauwelsstraße 30, 52074 Aachen, Germany

^3^Uniklinik RWTH Aachen, Institute of Gynecology and Obstetrics, Pauwelsstraße 30, 52074 Aachen, Germany

^4^Uniklinik RWTH Aachen, Electron Microscopy Facility, Institute of Pathology, Pauwelsstraße 30, 52074 Aachen, Germany

^5^Uniklinik RWTH Aachen, Institute for Occupational, Social and Environmental Medicine, Pauwelsstraße 30, 52074 Aachen, Germany

^6^Maastricht University Medical Center, Department of Radiology and Nuclear Medicine, P. Debeylaan 25, 6229 HX Maastricht, The Netherlands

^7^Center of Integrated Oncology Aachen Bonn Cologne Duesseldorf (CIO ABCD), Cologne, Germany

Corresponding authors: [jpena@ukaachen.de](mailto:jpena@ukaachen.de), [tlammers@ukaachen.de](mailto:tlammers@ukaachen.de)

**Figure S1:**

**Figure S1**. **Characterization of mono- or tri-DFO-functionalized [PEG-b-p(HPMAm-Bz)]-based block co-polymers.** **A)** ^1^H NMR (400 MHz, DMSO-d_6_) characterization and determination of degree of polymerization. **B)** GPC determination of molecular weight. **C)** Summary table of measured properties for the three polymers. Poly-0 corresponds to the medium polymer previously reported.^22^ (NB: DoP refers to degree of polymerization as determined by NMR).

**Figure S2:**

**Figure S2**. **Colloidal stability of polymeric micelles. A)** Critical micelle concentration (CMC) of the DFO-functionalized polymers (PolyDFO-1 and PolyDFO-3) as compared to the non-functionalized polymer (Poly-0) by fluorescence dilution of coumarin. **B)** Calculated CMC from piecewise fitted linear model. All three polymers demonstrate comparable CMC, indicating similar colloidal stability of the corresponding micelles upon dilution.

**Figure S3:**

**Figure S3. Stability of DFO-functionalized polymeric micelles over time. A-B)** Size (hydrodynamic diameter) (A) and PDI (B) of PM1 and PM3 micelles stored in buffered media (PBS) at 4°C, pH 7.4 for 1 month. **C-D)** Size (hydrodynamic diameter) (C) and PDI (D) of PM1 and PM3 micelles incubated in FBS at 37 °C, pH 7.4 for 96 hours.

**Figure S4:**

**Figure S4**. **DFO PET imaging performance.** Representative PET MIP of [^89^Zr]Zr-DFO injected 4T1-tumor bearing mice over time, 5 MBq intravenous injection via tail vein. Low tumor uptake and contrast are achieved, making tumor localization challenging. Increasing background noise after 4 hours indicates rapid tracer elimination.

**Figure S5:**

**Figure S5**. **Quantification of free DFO PET imaging contrast.** [^89^Zr]Zr-DFO PET imaging contrast over time after 5 MBq intravenous injection via tail vein. The high uptake in the kidneys and decay in imaging contrast after 4 hours in other organs like liver indicate rapid elimination of the tracer. N = 4 mice per group.

**Figure S6:**

**Figure S6**. **FDG PET imaging performance.** Representative dynamic PET MIP of [^18^F]FDG injected in 4T1-tumor bearing mice, 40-45 mins post-injection, 5 MBq intravenously via tail vein. Low target-to-background ratio is achieved in the tumor, thus complicating tumor localization with this tracer.

**Figure S7:**

**Figure S7. PET imaging performance.** Representative 2D PET/CT slices of [^89^Zr]Zr-PM1 injected into 4T1-tumor bearing mice over time, 5 MBq intravenous injection via tail vein.

**Figure S8:**

**Figure S8. Micelle tumor accumulation and biodistribution in a patient-derived TNBC xenograft model.** Ex vivo uptake of [^89^Zr]Zr-PM1 in a BCSC-1 patient-derived triple negative breast cancer models by gamma counter, normalized by tissue mass and relative to muscle as background (target-to-background ratio, TBR), 48 hours post-intravenous injection via tail vein. The similar biodistribution profile compared to that of the 4T1 model showcases applicability of the imageable micelle platform in different cancer models. N = 5 mice per group.

**Figure S9:**

**Figure S9. Ex vivo imaging of micelle tumor targeting heterogeneity.** Ex vivo autoradiography of **A)** 4T1 triple-negative breast cancer, and **B)** BCSC-1 triple negative breast cancer tumors, demonstrating both heterogeneity in the intratumoral distribution of [^89^Zr]Zr-PM1 in individual tumors, as well as the heterogeneity in micelle uptake in different cancer models.

**Figure S10:**

**Figure S10**. **Size comparison of theranostic micelles.** Size distribution by DLS of paclitaxel (PTX) drug-loaded polymeric micelles used for in vivo biodistribution of the theranostic formulations.

**Figure S11:**

**Figure S11. Comparison of drug-loading performance of the theranostic micelles.** Paclitaxel drug encapsulation efficiency and loading capacity of the DFO-functionalized polymeric micelles (PTX-PM1, PTX-Blend-PM and PTX-Cryo-Blend-PM), as compared to the non DFO-functionalized micelles (PTX-PM0). This demonstrates that the micelles have equivalent drug loading characteristics. N = 3 micelle formulations. Statistical analysis by one-way ANOVA: p < 0.05 (*), p < 0.01 (**), p < 0.001 (***).

**Figure S12:**

**Figure S12. Radiolabeling of theranostic micelles.** Final radiochemical purity of ^89^Zr-labeled drug-loaded polymeric micelles. Equivalent final radiochemical purity is achieved in all drug-loaded polymeric micelles, demonstrating that the density of DFO in the micelle core (at an average of one DFO/chain or below) does not impact the radiolabeling. N = 3 micelle formulations. Statistical analysis by one-way ANOVA: p < 0.05 (*), p < 0.01 (**), p < 0.001 (***).

**Figure S13:**

**Figure S13**. **Drug retention in theranostic polymeric micelles.** Paclitaxel (PTX) drug retention in ^89^Zr-radiolabeled polymeric micelles, demonstrating comparable drug-release kinetics from pure and blended DFO-micelles. N = 3 micelle formulations. Statistical analysis by two-way ANOVA with Bonferroni correction: p < 0.05 (*), p < 0.01 (**), p < 0.001 (***).

**Figure S14:**

**Figure S14**. **Ex vivo immunofluorescent staining of tumor tissues for macrophages and perfused blood vessels.** 10 µm tumor tissues were stained for nuclei (DAPI, blue) and tumor-associated macrophages (F4/80, green). 15 minutes before sacrifice, animals were injected with 60 µL of 5 mg/mL rhodamine–conjugated ricinus communis agglutinin I (Rhodamine-Lectin, red) to label perfused blood vessels. A representative field of view from each group is shown here. Scale bar = 50 µm.

**Figure S15:**

**Figure S15. Quantification of tumor microenvironment. A-B)** Mean F4/80 tumor-associated macrophage density in 4T1 tumors (A) and macrophage density in the core and periphery of the tumors (B). **C-D)** Mean perfused blood vessel density in 4T1 tumors (C) and perfused blood vessel density in the core and periphery of tumors (D). N ≥ 3 tumors/group, N = 5 fields of view for each core and periphery. Statistical analysis by one-way ANOVA with Bonferroni correction: p < 0.05 (*), p < 0.01 (**), p < 0.001 (***).
